# Successful multi-stage treatment of stoma limb perforation following Hartmann’s operation report a case

**DOI:** 10.1186/s40792-020-00827-8

**Published:** 2020-05-14

**Authors:** Jun Kataoka, Toshikatsu Nitta, Masato Ota, Yuko Takashima, Miyuki Imanishi, Kensuke Fujii, Takashi Ishibashi

**Affiliations:** 1 Department of Surgery, Gastroenterological Center, Shunjukai Shiroyama Hospital, 2-8-1 Habikino, Habikino city, Osaka, 583-0872 Japan; 2 Department of Internal Medicine, Gastroenterological Center, Shunjukai Shiroyama Hospital, Habikino city, Osaka, Japan; 3 Department of General and Gastroenterological Surgery, Osaka Medical College Hospital, Takatsuki, Osaka, Japan

**Keywords:** Stoma limb perforation, Colostomy, Abdominal wall abscess

## Abstract

**Background:**

Stoma-related complications are not rare, whereas the spontaneous perforation of the stoma limb is relatively rare. Herein, we report a case of stoma limb perforation which occurred after Hartmann’s operation.

**Case presentation:**

A 50-year-old Japanese man presented to our Hospital with acute and severe abdominal pain. Abdominal computed tomography (CT) scan revealed that an abscess with free air was formed around the sigmoid colon. We performed Hartmann’s operation, whereas he experienced redness, purulent discharge, and swelling around the colostomy at 10 days postoperatively. The contrast-enhanced CT scan of the abdomen revealed an abscess formation with air around the colostomy. He was diagnosed with an abdominal wall abscess due to perforation of the stoma limb.

After the drainage, his symptoms were ameliorated by oral analgesics, anti-inflammatory drugs, and prophylactic antibiotic. Four months after the first operation, we performed a closedown of the sigmoid colostomy and fistula resection. The patient’s postoperative course was uneventful, and he was discharged 14 days later.

**Conclusions:**

This case depicts rare complications of Hartmann’s operation. Operation is usually performed in patients with stoma limb perforation. However, if they are stable and the abscess is located in their abdominal wall, they may be treated successfully using a multi-stage approach of local drainage toward the stoma wall followed by stoma closure.

## Background

Stoma-related complications are with a frequency of 10–70%. Peristomal dermatitis, parastomal hernia, stomal prolapse, and subsidence are common, whereas the spontaneous perforation of the stoma limb is relatively rare [1, 2]. The prognosis in colon perforation is generally unfavorable; however, there is no standard treatment for stoma limb perforation. A multi-stage or elective operative treatment should be considered when the abscess is localized to the abdominal wall, local drainage is likely to be effective, the colostomy is not necrotic, and the patient’s condition is stable. Herein, we report a case of stoma limb perforation that occurred after Hartmann’s operation.

## Case presentation

A 50-year-old Japanese man presented to the Department of the Gastroenterological Center at Shunjukai Shiroyama Hospital with acute and severe abdominal pain.

An abdominal computed tomography (CT) scan revealed that an abscess with free air was formed around the sigmoid colon. Therefore, the patient was admitted to our hospital for operative treatment of the perforated sigmoid colon due to diverticulitis. Hartmann’s procedure was performed under general anesthesia with the patient in the supine position. The total operating time was 188 min, and the intraoperative blood loss was 200 ml.

**Fig. 1:**
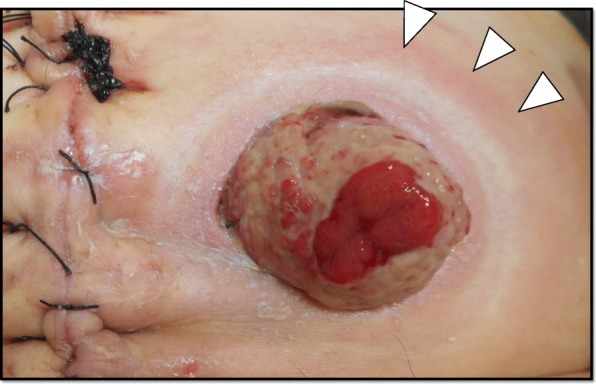
Slight redness and swelling at the colostomy site (white arrows)

**Fig. 2:**
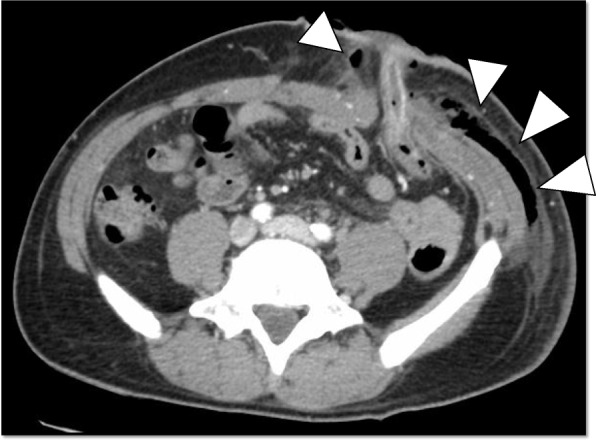
Initial computed tomography of the abdomen revealed an abscess formation with air around the colostomy (white arrows)

The patient’s postoperative course was uneventful for several days. He resumed oral intake at 2 days postoperatively, and his first defecation from colostomy was at 5 days postoperatively. Subsequently, he experienced mild redness, purulent discharge, and swelling around the colostomy at 10 days postoperatively (Fig. 1). Routine blood tests showed that his white blood cell count was 20,900/mm^3^, and the C-reactive protein (CRP) level was 10.12 mg/dl (Table 1). The contrast-enhanced CT scan of the abdomen revealed an abscess formation with air around the colostomy (Fig. 2).

**Table 1:**
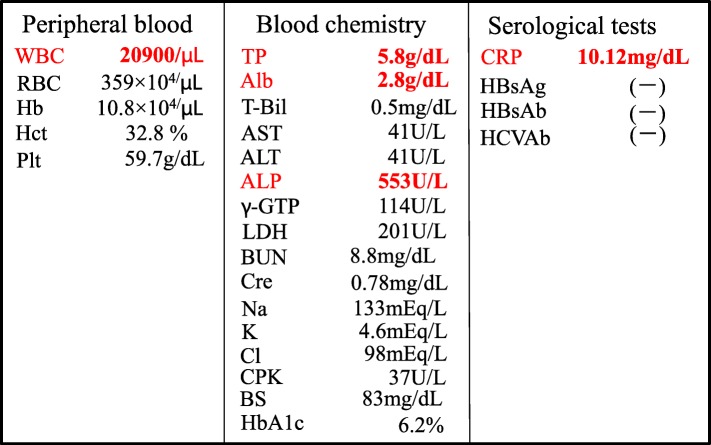
Laboratory findings. Routine blood test showed his white blood cell count was 20,900/mm^3^ and C-reactive protein (CRP) was 10.12 mg/dl

**Fig. 3:**
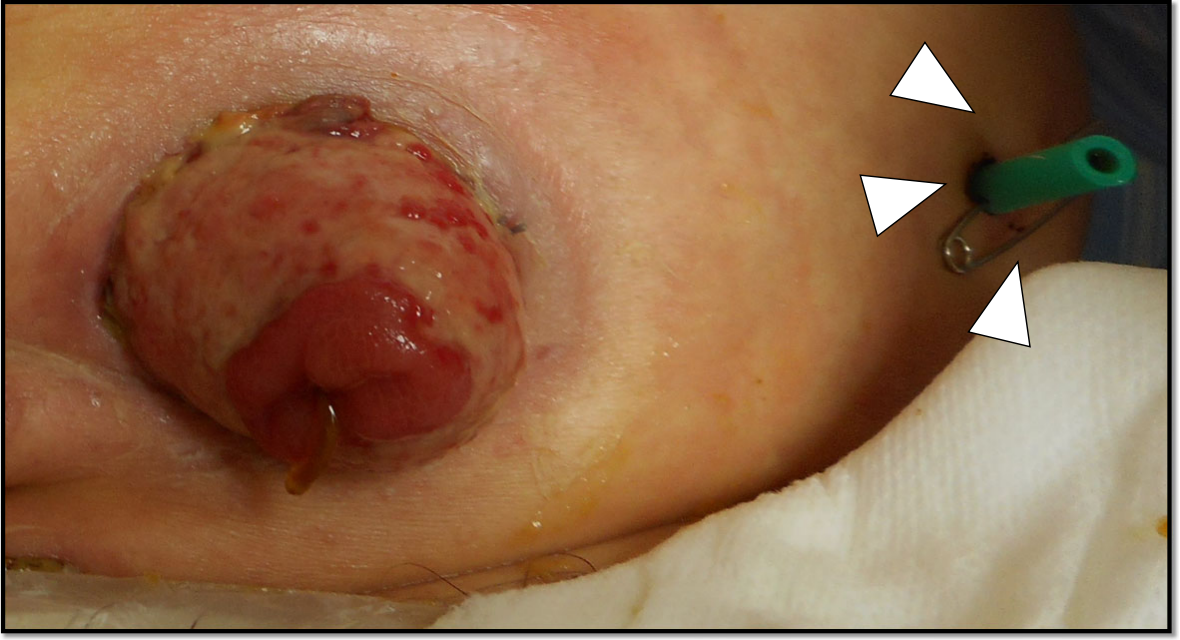
We immediately performed the drainage of the abscess from the left lower quadrant abdominal wall laterally near the colostomy and indwelled an 18-Fr silicon drain tube for 8 days

The patient was diagnosed with an abdominal wall abscess due to perforation of the stoma limb. We immediately performed drainage of the abscess from the left lower quadrant abdominal wall laterally near the colostomy and indwelled an 18-Fr silicon drain tube for 8 days (Fig. 3).

**Fig. 4:**
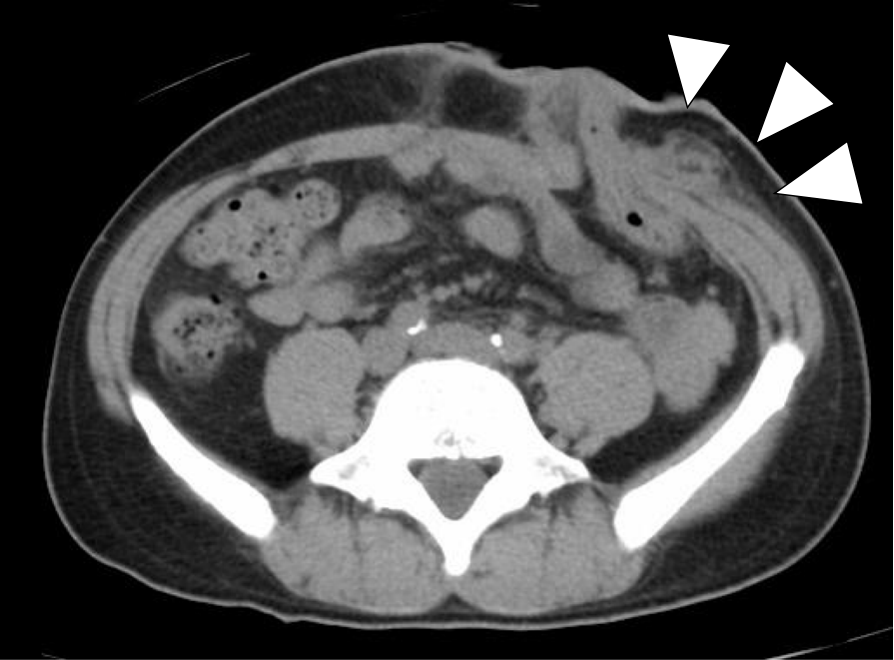
Follow-up computed tomography of the abdomen after the drainage showed abscess decrease (white arrows)

**Fig. 5:**
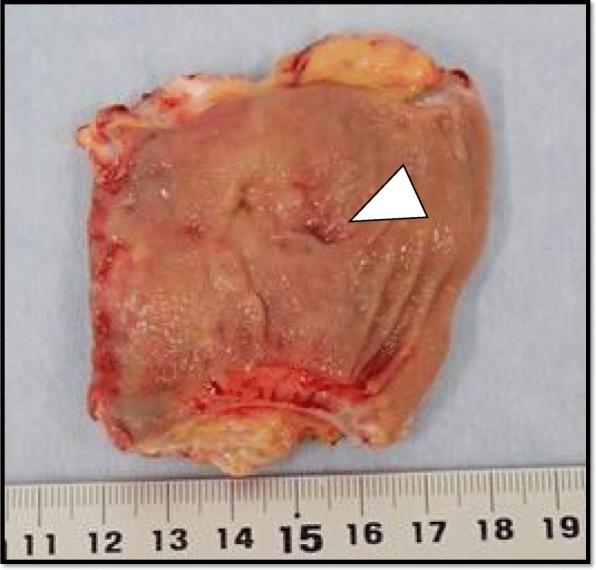
The specimen was not malignant and a 0.5 cm × 0.5 cm perforation hole in the mesenteric side of the stoma limb was seen

After the drainage, his symptoms were ameliorated by oral analgesics, anti-inflammatory drugs, and prophylactic antibiotics. A follow-up CT scan at 29 days postoperatively showed that the abscess had decreased (Fig. 4). Four months after the first operation, his vital signs and symptoms were relatively stable. We performed a closedown of the sigmoid colostomy and fistula resection as the second-stage surgery. The specimen was not malignant and a 0.5 cm × 0.5 cm perforation hole was seen in the mesenteric side of the stoma limb (Fig. 5). The total operating time was 235 min, and the intraoperative blood loss was 110 ml. The patient’s postoperative course was uneventful, and he was discharged 14 days later.

## Discussion

The complications associated with stoma occur at a frequency of 10–70%. The most common complications are peristomal dermatitis, parastomal hernia, stomal prolapse, and subsidence; in contrast, the perforation of the stoma limb is relatively rare [1, 2] and reportedly develops most often in patients with chronic constipation or trauma or in the early postoperative phase of colostomy or in those taking medications such as opioids or tricyclic antidepressants [3].

We searched for case reports published from April 1995 to December 2019 in the Japan Medical Abstracts Society and PubMed database using the keywords “spontaneous perforation” or “stoma limb perforation.” The search resulted in nine cases, including ours (Table 2) [3–13]. Colectomy and revision of stoma (including stoma closure) were chosen as the treatment methods in eight cases. In comparison, the treatment in the other case included two-stage elective surgery after drainage. We also selected the same treatment course for managing our patient.

**Table 2:** Search results of published case reports from April 1995 to December 2019 in the Japan Medical Abstracts Society and in PubMed database using the keywords “spontaneous perforation” or “stoma limb perforation”

Year	Authors	Age/sex	Stoma site	Factor	Perforation site	Operation	Hospital stay
1995	Sakamoto et al. [4]	37/M	Sigmoid colon	Trauma	3 cm oral side	Colectomy + revision of stoma	14
2009	Tamura et al. [5]	83/F	Sigmoid colon	Stercoral	Stoma limb	Colectomy + revision of stoma	26
2010	Ikenishi et al. [6]	78/F	Sigmoid colon	Stercoral	Stoma limb	Colectomy + revision of stoma	17
2011	Ikari et al. [7]	80/M	Sigmoid colon	Cancer	Stoma limb	Colectomy + revision of stoma	41
2011	Hata et al. [8]	52/F	Transverse colon	Stercoral	5 cm oral side	Colectomy + revision of stoma	129
2013	Ozawa et al. [9]	69/F	Sigmoid colon	Trauma	Stoma limb	(1) Drainage, (2) colectomy + revision of stoma	(1) 9, (2) 12
2013	Kim et al. [10]	70/M	Sigmoid colon	Stercoral	Stoma limb	Colectomy + revision of stoma	32
2013	Kim et al. [10]	71/M	Sigmoid colon	Stercoral	Stoma limb	Drainage, direct suture + colectomy + revision of stoma	44
2015	Harada et al. [11]	69/M	Sigmoid colon	Trauma	10 cm oral side	Colectomy + revision of stoma	27
2016	Fukuoka et al. [12]	61/M	Sigmoid colon	Stercoral	Stoma limb	Colectomy + revision of stoma	14
2016	Ikegami et al. [3]	78/M	Sigmoid colon	Stercoral	Stoma limb	Colectomy	N/A
2017	Iwata et al. [13]	83/F	Sigmoid colon	Stercoral	Stoma limb	Colectomy + revision of stoma	97
2019	Our case	50/M	Sigmoid colon	Spontaneous	Stoma limb	(1) Drainage, (2) colectomy + stoma close	(1) 41, (2) 14

Spontaneous perforation often occurs at the sigmoid or rectosigmoid colon site due to poorer distensibility, narrower diameter, and slower transit time in these regions. A colostomy is frequently created with the sigmoid colon, which has more mobility than other colonic regions and is susceptible to spontaneous colon perforation.

Spontaneous perforation of the colon is a rare condition secondary to trauma, malignancy, iatrogenic disease, diverticulum, and inflammatory bowel disease. There are macroscopic and histological criteria as diagnostic criteria for this disease [14, 15]; the macroscopic criteria were modified as follows (1) there is no macroscopic lesion in the perforated bowel wall, (2) there is no gastrointestinal foreign body or obstruction, (3) there are no intraperitoneal abnormalities including adhesions and internal hernia or ventral hernia, and (4) it can be denied that there is intestinal injury by direct external force to the bowel and medical practice. The histological criteria were modified as follows (1) there is no mucosal rupture and invasion into the serosa in the peripheral edge, (2) there is a rupture in the muscular layer and the stump is relatively sharp, and (3) there is no abscess and granulation although the histological finding showed acute or subacute inflammation.

The etiology of spontaneous colon perforation includes the following: (1) laceration and thinning in the bowel wall caused when dehydrated fecaloma passes through the colon, (2) a disorder of circumstance with hyperextension bowel wall, and (3) inordinate pressure applied to the fragile bowel wall [16, 17]. It is important to diagnose the factors contributing to colon perforation, whereas it is not easy to distinguish between stercoral or spontaneous colon perforation in clinical practice. As the diagnostic criteria in stercoral perforation, Huttunen et al. reported that in a perforated stercoraceous ulcer, the perforation was a round or an ovoid hole with necrotic and inflammatory edges; however, in the idiopathic form, the perforation was a tear with a normal appearance of the colonic wall without being involved in the diverticulum [18, 19], and Maurer et al. presented the criteria of stercoral perforation, which includes the following: (1) round or ovoid perforation, > 1 cm in diameter; (2) fecalomas present within the colon, protruding through the perforation site or lying within the abdominal cavity; and (3) pressure necrosis or ulcer and chronic inflammatory reaction around the perforation site seen microscopically [20].

Prognosis in colon perforation is generally unfavorable although improvements in diagnostic technology and treatment have resulted in reducing the mortality rates. However, there is no standard treatment for stoma limb perforation. Surgical treatment for abscess draining is performed regardless of a patient’s clinical, laboratory, and radiological findings. When emergency procedures are not warranted, a multi-stage or elective operative treatment algorithm may be more effective [9]. This course should be considered when the abscess is localized to the abdominal wall, local drainage is likely to be effective, the colostomy is not necrotic, and the patient’s condition is stable.

We diagnosed our case with spontaneous perforation of the colon because there was no macroscopic lesion, injury, and dehydrated fecaloma in the bowel and abdominal cavity. This is in line with the criteria of spontaneous perforation. Moreover, it is likely the stoma limb was weakened and perforated due to the poor condition of the colon wall coincident to increased bowel pressure. As the patient was in a stable condition and the abscess well-localized, we were able to perform the local drainage toward the wall around the stoma. The follow-up period after the local drainage was uneventful; therefore, we performed perforation excision and stoma closure instead of reconstruction of the colostomy.

## Conclusion

In conclusion, we presented a case with a rare complication of Hartmann’s operation. Patients with stoma limb perforation are usually treated operatively. However, if their general status is stable and the abscess is located in their abdominal wall, the patient may be treated successfully with a multi-stage approach of local drainage toward the wall followed by stoma closure.

## Data Availability

Not applicable

## Abbreviations

CRP: C-reactive protein
CT: Computed tomography
WBC: White blood count
